# Bayesian shrinkage mapping of quantitative trait loci in variance component models

**DOI:** 10.1186/1471-2156-11-30

**Published:** 2010-04-29

**Authors:** Ming Fang

**Affiliations:** 1 Life Science College, Heilongjiang August First Land Reclamation University, Daqing, 163319, China; 2 Department of Animal Genetics and breeding, College of Animal Science and Technology, China Agricultural University, Beijing 100193, China

## Abstract

**Background:**

In this article, I propose a model-selection-free method to map multiple quantitative trait loci (QTL) in variance component model, which is useful in outbred populations. The new method can estimate the variance of zero-effect QTL infinitely to zero, but nearly unbiased for non-zero-effect QTL. It is analogous to Xu's Bayesian shrinkage estimation method, but his method is based on allelic substitution model, while the new method is based on the variance component models.

**Results:**

Extensive simulation experiments were conducted to investigate the performance of the proposed method. The results showed that the proposed method was efficient in mapping multiple QTL simultaneously, and moreover it was more competitive than the reversible jump MCMC (RJMCMC) method and may even out-perform it.

**Conclusions:**

The newly developed Bayesian shrinkage method is very efficient and powerful for mapping multiple QTL in outbred populations.

## Background

There are two kinds of models which can be used to map QTL in outbred populations, the allelic substitution model [1-3] and the variance component model [4-7]. In the allelic substitution model, the number of QTL alleles is assumed to be known, and the QTL allelic substitution is estimated by the given linkage phases of parents, which can be inferred from genotypes of family members. The least square [2] and maximum likelihood [1,3,8] of interval mapping are two popular statistical approaches for such models. Compared with the allelic substitute model, the variance component model is more robust because it can handle an arbitrary number of alleles with arbitrary modes of gene actions[9]. Moreover, the linkage phase of parents is unnecessary, which is nice since it is hard to accurately infer, particularly when family size is small, such as with human populations. Therefore, the variance component model is usually used to map QTL in outbred populations [4-7,9-12]. In the variance component model, the identity-based-decent (IBD) matrix may be different for each locus and can provide information to localize the QTL. The least square method [10,11] and the maximum likelihood method [4,13] are also two important statistical methods for handling this model.

Because of the polygenic nature of quantitative traits, multiple QTL mapping is a problem of model selection. The least square method and the maximum likelihood method can nicely handle single QTL model, but is difficult for them to handle multiple QTL model. Recently, the Bayesian reversible jump MCMC (RJMCMC) method has been used to map multiple QTL in the variance component model [9,12]. However, it still has some disadvantages. Because the model dimension is variable, it usually has poor mixing character and is difficult to converge [14-16]; moreover, it is also difficult to explore all the model space, especially in genome-wide mapping where thousands of possible locus are scanned [16].

Therefore, in this article I proposed a model-dimension-fixed method, in which the estimate of variance is very precise for nonzero-effect QTL, and gradually converges to zero for zero-effect QTL. Therefore, special model selection is needless. It is similar to the recent Bayesian shrinkage estimation methods [15,17-19], which are based on the allelic substitution model, whereas my method is based on the variance component model. The efficiency of the new method is demonstrated by a series of simulation experiments.

## Method

### Genetic model

Suppose that one has a sample of *n *individuals from outbred populations. Assuming that QTL dominant effect and polygenic dominant effect are absent. Then the linear model can be expressed as(1)

where, **y **is the *n *× 1 phenotypic vector; **β **is the *k *× 1 vector of covariate effects; *k *is the number of the covariate; **X **is the *n *× *k *design matrix related to the covariate effects; **a**_*j *_~ *N*(**0**, **Φ**_*j*_) is the *n *× 1 vector of random QTL effect, for *j *= 1,2, ..., *q*, where **Θ**_*j *_is the IBD matrix and can be inferred by the conditional expectation approach [20]; and  is the QTL variance; **e **~ *N *(**0**, **I**_*n*_) is the vector of random error, where **I**_*n*_, is the *n *× *n *identity matrix and  is the residual variance; *q *is the maximum QTL number, which is set beforehand; g ~ *N*(**0**, **A**) is the *n *× 1 vector of random polygenic effect, here **A **is the additive relationship matrix and  is the polygenic additive variance, the polygenic term **g **may be excluded from equation (1) in genome-wide mapping. The variance component model can be expressed as(2)

Similarly, the term of polygenic variance **A** should also be excluded from equation (2) in genome-wide mapping.

### Prior specification and joint posterior distribution

Yi and Xu [9] assigned a uniform prior distribution for QTL variance, , but in my method, the Jeffreys' hyper prior  is assumed. The special prior is the key in the new method and will be illustrated in detail later. The prior for polygenic variance and residual variance is assumed to follow scaled inverted chi-square distribution with degree of freedom **ω **and scaled parameter *s*^2 ^(see also [21] for detail); and the prior for covariant effect and QTL position *λ*_*j *_are assumed to follow normal distribution, **β **~ *N *(**β**_0_, **V**_0_), and uniform distribution, respectively. The joint posterior distribution is given in Appendix.

### Updating QTL variance by random walk Metropolis-Hastings algorithm

Because there is no close form for the posterior distribution of QTL variance , the Metropolis-Hastings algorithm [22,23] is used to simulate it. I firstly propose a new QTL variance and then accept it according to its acceptance probability.

#### Generating the new proposal QTL variance

I employ the Browne's method [24], a special random walk Metropolis-Hastings algorithm (RWM-H) to update QTL variance. Firstly a new QTL variance  is proposed and sampled from a scaled inverted chi-squared distribution, conditional on the current value of QTL variance , , with the degree of freedom ν and the scaled parameter  that equals the expectation of the current value , *i.e. *, and then the new QTL variance is accepted according to its accept probability. Since the new generated value closely relies on the old one, this approach is a special case of RWM-H, and the degree of freedom ν is equivalent to the tuning parameter [21,24].

#### *Calculating the acceptance probability*

The new proposal QTL variance is accepted with probability equal to min (1, *r*), where,(3)

and  represents all elements of **θ **except . In equation (3), the first term is likelihood, the second is *prior *and the third is called proposal ratio or Hastings ratio [23]. Because the proposal distribution is not symmetric, , *hr *must be computed, and(4)

### MCMC implementations

The implementations of the MCMC algorithm are summarized as follows:

a. Initialize all parameters from legal values;

b. Update the covariate effect **β**;

c. Update the QTL variance ;

e. Update the polygenic variance ;

f. Update the residual variance ;

g. Update the QTL positions .

The covariate effect **β **is updated by the efficient Gibbs sampler; the updating approach of  and  are similar to that of  (see also [21] for detail); the updating of the QTL position  is illustrated in Appendix.

### Post-MCMC analysis

To summarize the posterior probability, I divide the genome into bins with interval of 1 cM and calculate weighted QTL variance for each bin. The weighted QTL variance is defined as the estimate of the QTL variance multiplied by its posterior probability at each bin (see also [21]), which is the modification of the weighted QTL effect [15]. If the profile of the weighted QTL variance generates a notable bump on the genome, the QTL is claimed as detected [15,19,25].

## Results

I simulated 500 independent full-sib families with 6 individuals in each one, and therefore 3,000 individuals were investigated in my study. The parents of the full-sib families were randomly sampled from a large outbred populations in Hardy-Weinberg and linkage equilibrium. One chromosome with the length of 100 cM was simulated, and 11 evenly spaced markers covered the chromosomes with an average marker interval of 10 cM. I assigned 6 alleles for each marker and infinite alleles for each QTL. Three QTL were simulated on the genome positioned at 15 cM, 45 cM and 75 cM, respectively. The additive variances of the three QTL were respectively 0.5, 1.2 and 0.8, and the dominant variances were assumed absent. The residual effect for each offspring was randomly sampled from normal distribution with mean 0 and variance  = 1.0. The polygenic variance  = 1.0. The simulation method for the polygenic effect has been illustrated in [26]. The population mean equaled to 0. The phenotypic value for each sib was the sum of population mean, QTL effects, polygenic effect and residual effect. Therefore, the heritabilities explained by the three QTL were respectively 11.1%, 26.7% and 17.8%.

Before performing the simulated experiments, I firstly gave a default setup. The excepted QTL number *q*_0 _= 2, which may lead to the maximum QTL number,  (see [27] for detail); the degree of freedom for generating the proposal variance ν = ν_*A *_= ν_*e *_= 10; the hyper-parameter ω_*A *_= ω_*e *_= 3 and  (are approximately estimated as the phenotypic variance), and hence  (equal to the expectation of their variance estimations, see [21]). Because ω_*A *_and ω_*e *_took a small value, the values of  and  would have ignorable effect on their estimates. The importance of the hyper-parameter also has been illustrated in [21]. The MCMC ran for 21,000 rounds and the data was saved with every 10 rounds after the first 1,000 MCMC was discarded, so that there were 2,000 (20,000/10) posterior samples for posterior analysis.

### Performance on simulated data with zero-QTL model

**Figure 1:**
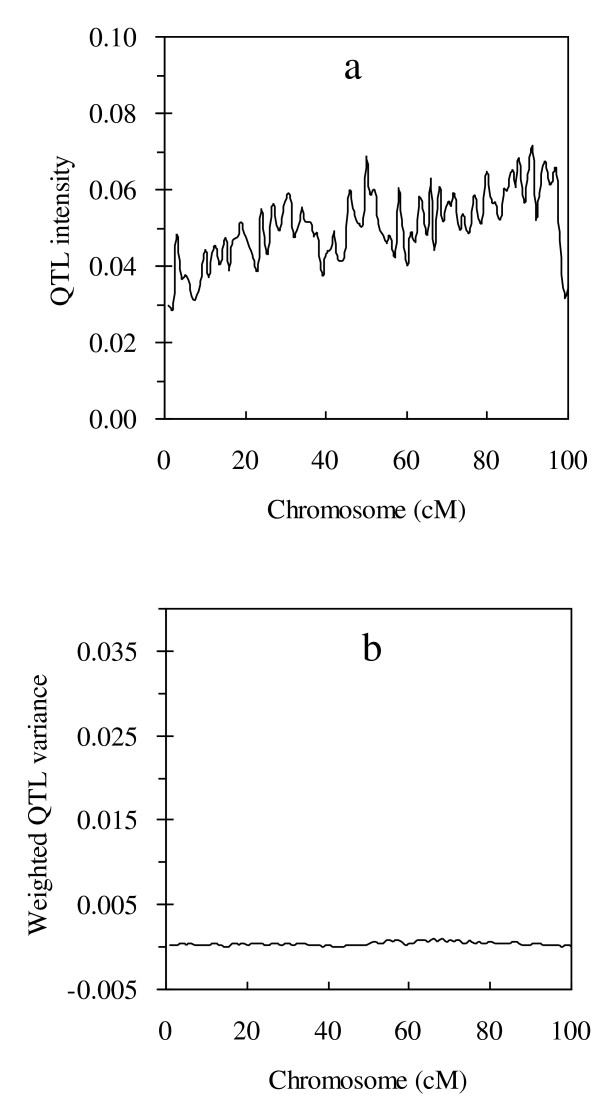
**Profiles of QTL intensity (a) and weighted QTL variance (b) obtained from the proposed method under simulated zero-QTL model**.

**Figure 2:**
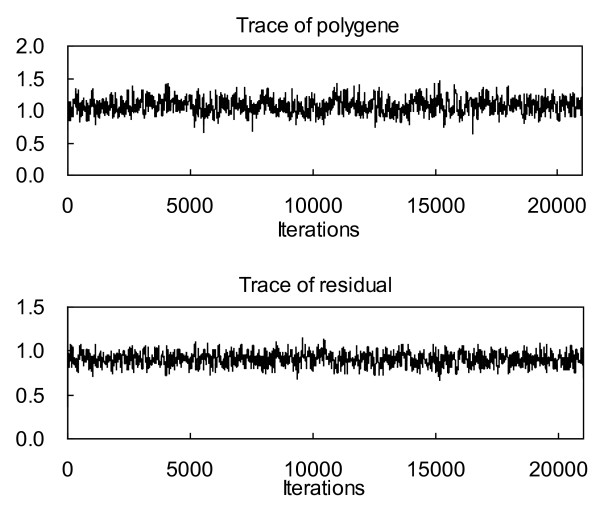
**Traces of polygenic variance and residual variance obtained from the proposed method**.

To demonstrate the special character of the proposed method, I firstly analyzed the data from the simulated zero-QTL model. The profiles of QTL intensity and weighted QTL variance are plotted in Figure 1a and Figure 1b, respectively. The profile of weighted QTL variance gives very noisy signals for QTL detection, but the values of weighted QTL variance are very tiny and the profile is much flat, which reflects that the proposed method can effectively shrink the values of the variance of zero-effect QTL infinitely close to zero. Figure 2 gives the MCMC traces of the polygenic variance and the residual variance, and indicates that the estimates of them are all close to their true values, 1; moreover, the Markov chains of them converge fast and mix well.

### Investigation into the performance of the special RWM-H algorithm

**Figure 3:**
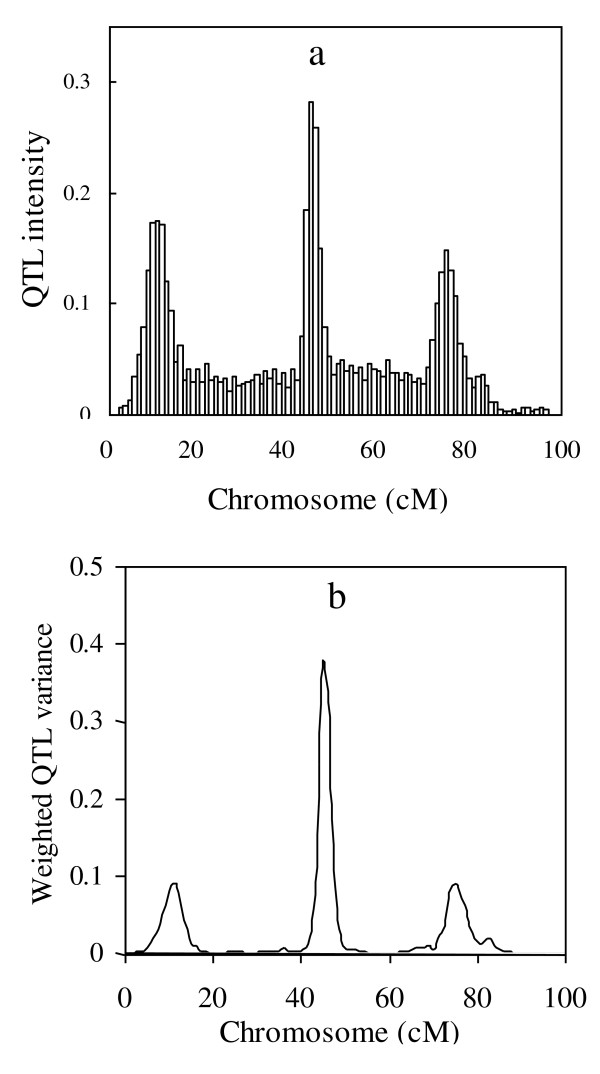
**Typical profiles of QTL intensity (a) and weighted QTL variance (b) from the proposed method**.

**Figure 4:**
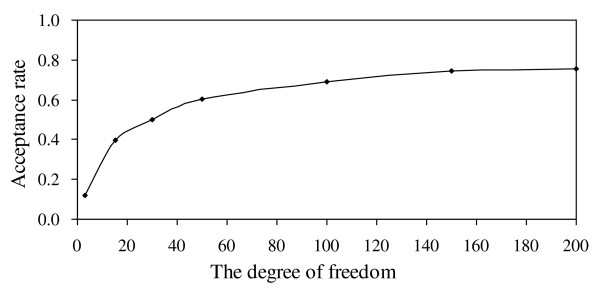
**The change of the acceptance rate against the degree of freedom by the proposed method**.

I use a special RWM-H algorithm to update the variance components, and the new proposal variance σ^2 ^(QTL variance, polygenic variance or residual variance) is sampled from the scaled inverted chi-squared distribution with degree of freedom ν and scaled parameter the variance of the current round. In order to test the influence of ν, I set ν as 3, 15, 30, 50, 100, 150 and 200, respectively. The QTL intensity histogram [28] is plotted in Figure 3a. There are three peaks bumped on the chromosome, but QTL intensity is not used in QTL detection. I also plot the profile of weighted QTL variance, and the general pattern is given in Figure 3b. All other experiments have performed similar pattern, so the figures are not shown. I find the profile of weighted QTL variance is rather flat for the positions that have no QTL, which makes the signals of QTL clearer than QTL intensity. The parameter estimates are listed in Table 1, and there are no clear differences in parameter and standard deviation estimates for different ν. Furthermore, I summarized the acceptance rate of the M-H sampler for the variance components. Because it is cumbersome to show them separately, I averaged the acceptance rate over all variance components under different setting of ν. I further plot the profile of the change of the acceptance rate against ν in Figure 4. It shows that the acceptance rate increases by ν, but the rate of change decease by ν. When ν is smaller than 30, the curve is much steeper, but it flatten when ν is larger than 30. The degree of freedom ν may influence the acceptance rate in the special RWM-H algorithm, and hence it is equivalent to the tuning parameter in the traditional Metropolis-Hastings algorithm. Finally, I found that when ν is larger than 200, the shrinkage character is hardly held. The reasons will be addressed in Discussion.

**Table 1:** The estimates of the QTL parameters and their standard deviations obtained from the proposed method under different levels of degree of freedom.

d.f.	Position			QTL variance			Population mean	Polygenic variance	Residual variance
					
	QTL1	QTL 2	QTL 3	QTL 1	QTL2	QTL3			
**3**	11	45	75	0.827 (0.363)	1.314 (0.232)	0.592 (0.210)	0.029 (0.003)	0.827 (0.363)	1.164 (1.181)
**15**	11	45	75	0.481 (0.178)	1.358 (0.198)	0.584 (0.247)	0.028 (0.005)	0.812 (0.373)	1.182 (0.074)
**30**	11	45	75	0.508 (0.186)	1.329 (0.241)	0.602 (0.222)	0.034 (0.004)	0.849 (0.380)	1.151 (0.178)
**50**	10	45	75	0.784 (0.376)	1.333 (0.214)	0.635 (0.175)	0.031 (0.004)	0.784 (0.376)	1.181 (0.173)
**100**	10	45	74	0.834 (0.364)	1.353 (0.217)	0.637 (0.203)	0.036 (0.003)	0.834 (0.364)	1.162 (0.172)
**150**	10	45	76	0.786 (0.336)	1.356 (0.242)	0.647 (0.154)	0.030 (0.003)	0.786 (0.336)	1.178 (0.159)
**200**	10	46	76	0.875 (0.381)	1.232 (0.288)	0.582 (0.186)	0.029 (0.005)	0.87 (0.381)	1.140 (0.176)

The standard deviations are given in parentheses.

### Comparison with the regression method

**Figure 5:**
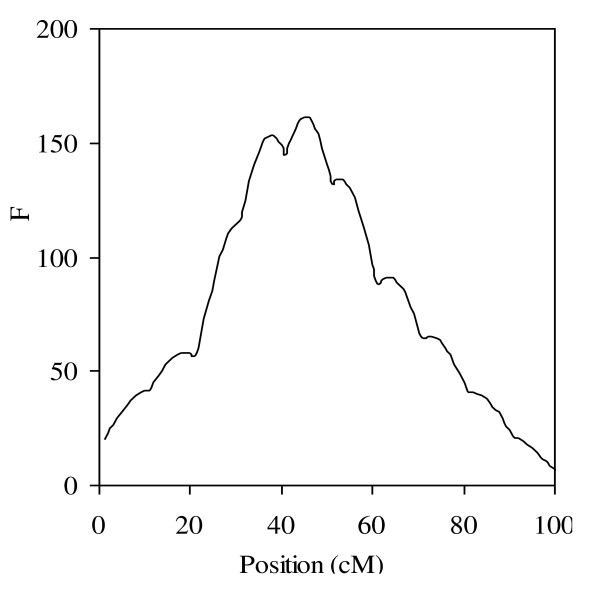
**Profiles of *F *statistic obtained from QTL Express**.

I also used the software QTL Express [29], an IBD based regression method, to analyze the simulated data. The method is based on single-QTL model. The profile of *F *statistic is plotted in Figure 5. Only one QTL localized at 44 cM was detected, and the simulated three QTL were combined together. However, the three QTL can be separated successfully by the new method. The results clearly reflect the advantage of the new method that uses multiple QTL model over the regression method that bases on single QTL model.

### Comparison with the RJMCMC method with repeat experiments

To compare the proposed method with the RJMCMC based method [9], I simulated 30 sets of data. For the RJMCMC method, the maximum QTL number and the expected QTL number were also the same as the default setup; the prior distribution of the QTL variance, polygenic variance and residual variance followed uniform distribution with endpoint being zero and phenotypic variance; the thinning interval was empirically set as 10; the burn-in period was 1,000 and the length of the complete chain was 201,000, and hence, there were 10,000 samples saved for posterior analysis. It took ~ 5 hr for the new and RJMCMC method on a Pentium IV PC with a 2.60-GHz processor and 1.00 GB RAM.

I list the empirical statistical power and the average estimates of 30 replications for both methods in Table 2, and the results show that: (1) the QTL detecting powers of the proposed method are slightly higher than that of the RJMCMC method; (2) there are no clear differences between the two methods in parameter estimates, and both are very precise.

**Table 2:** The average estimates of the QTL parameters from 30 replicated experiments in the proposed and RJMCMC method.

QTL No.	Power (%)	Position	QTL variance	Population mean	Polygenic variance	Residual variance
**Shrinkage analysis:**						
**QTL1**	90.0 (27/30)	13.7(3.40)	0.465(0.125)	0.028 (0.004)	1.118(0.324)	0.923(0.187)
**QTL2**	100.0(30/30)	45.8(1.32)	1.210(0.153)			
**QTL3**	96.7(29/30)	74.2(2.86)	0.787(0.116)			

**RJMCMC:**						
**QTL1**	86.7 (26/30)	14.1(3.98)	0.511(0.132)	0.031 (0.003)	1.201(0.315)	0.915(0.188)
**QTL2**	100.0 (30/30)	46.1(1.37)	1.150(0.140)			
**QTL3**	90.0(27/30)	74.9(3.51)	0.834(0.126)			

### Application in genome-wide mapping

In the genome-wide mapping, I simulated a large genome of 2,000 cM, covered by 201 evenly spaced markers with interval 10 cM. Five QTL were simulated with positions and effects in Table 3. The total heritability was 69.4%, and the heritability explained by these QTL ranged from 8.1% to 22.4%. The residual variance was 1.5 and the polygenic variance was 0. The family structure and other parameters were the same as the previous simulation.

**Table 3:** QTL parameters and their estimates obtained from the two methods in the genome-wide mapping.

QTL No.	True parameters		Estimations (new method)		Estimations (RJMCMC method)	
			
	Position(cM)	Variance	Position(cM)	Variance	Position(cM)	Variance
**1**	215	0.4	224	0.460(0.176)	224	0.539(0.131)
**2**	422	0.6	433	0.345(0.151)	433	0.457(0.120)
**3**	853	1.1	853	1.406(0.177)	854	1.502(0.190)
**4**	1450	0.5	1438	0.372(0.158)	1436	0.522(0.147)
**5**	1843	0.8	1844	1.138(0.182)	1844	1.224(0.174)

### Comparison with RJMCMC

The simulated data was analyzed with the proposed method and the RJMCMC method. In the proposed method, the excepted QTL number *q*_0 _= 3, which results the maximum QTL number *q *= 8; the degree of freedom generating the proposal variance ν = ν_*A *_= ν_*e *_= 10; the hyper-parameter ω_*A *_= ω_*e *_= 3 and  for the polygenic variance and the residual variance. The MCMC ran for 51,000 rounds and the data was saved with every 10 rounds after the first 1,000 MCMC was discarded, so that there were 5,000 (50,000/10) samples for posterior analysis. In the RJMCMC method, also *q*_0 _= 3 and *q*_*m *_= 8, thinning interval 10 and burn-in length 1,000, but the length of the complete chain was 201,000. In the genome-wide mapping, for all the QTL that affect the trait are included in the model, the polygenic variance is excluded from the model and thus needn't be estimated.

**Figure 6:**
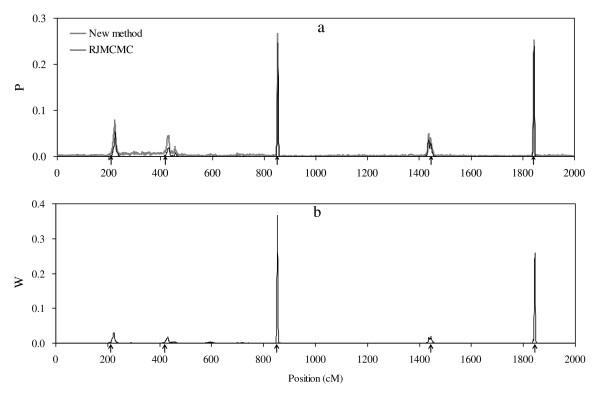
**Profiles of QTL intensity (a) and weighted QTL variance (b) in the genome-wide mapping**. The true locations of the simulated QTL are indicated by upward arrows on the horizontal axis.

**Figure 7:**
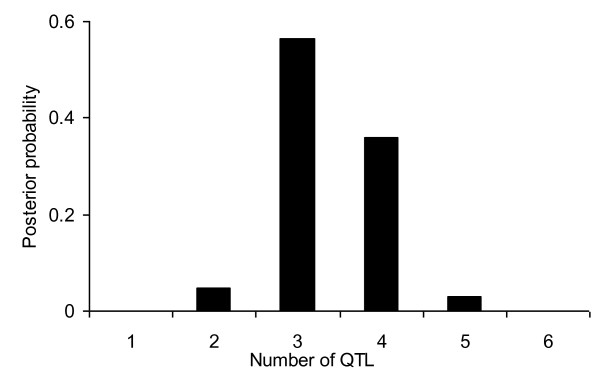
**Posterior distribution of the number of QTL from the RJMCMC method**.

The QTL intensity profiles of both methods are plotted in Figure 6a. It shows that the profile of the new method is higher than that of the RJMCMC method. But it is not sufficient to prove that the new method is more powerful than the RJMCMC method, because the QTL intensity is not used to detect QTL in shrinkage method. The profile of the weighted QTL variance is given in Figure 6b, and five clear bumps are found around their true simulated positions, which shows that the five simulated QTL are all detected by the new method. However, in the RJMCMC method, the estimated average number of QTL equaled to 3.37. The profile of the posterior QTL intensity is depicted in Figure 7, showing that the trait is mostly affected by three or four QTL with probability 0.565 or 0.359, and the estimated number of QTL is clearly smaller than the true number of QTL. The results suggest that my new method is competitive with the RJMCMC method, and may even out-perform it. The computing time of the proposed method and the RJMCMC method were nearly equal and they took ~ 24 hr on a Pentium IV PC with a 2.60-GHz processor and 1.00 GB RAM.

### Test on the sensitive of the maximum QTL number

**Figure 8:**
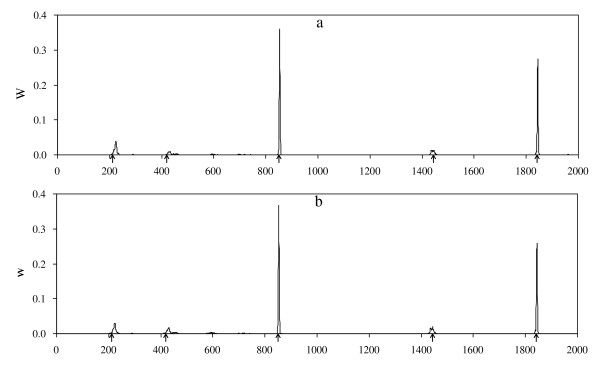
**Profiles of weighted QTL variance for *q *= 11 (a) and *q *= 14 (b) in the genome-wide mapping**. The true locations of the simulated QTL are indicated by upward arrows on the horizontal axis.

The maximum QTL number *q *is a hyper-parameter, which should be ascertained beforehand. In my study I followed the approach of [27] to ascertain it and . For testing the sensitive of the maximum *q*, I set the expected QTL number *q*_0 _= 4 and 5, which led to *q *= 11 and 14, respectively. The profiles of weighted QTL variance are plotted in Figure 8, and show no clear differences; moreover, they are very similar to the profile in Figure 6b that uses *q*_0 _= 3 and *q *= 8. The results demonstrated that the new method was not very sensitive to the value of the expected QTL number. I also ran the RJMCMC method under *q*_0 _= 4 and 5, and the estimated QTL numbers were 3.42 and 3.46, respectively. Clearly, they were also smaller than the true values.

## Discussion

The Jeffreys' hyper prior for QTL variance, , which is much crucial in Bayesian shrinkage analysis for inbred line crosses [15,17,18], is also the key to the new method. Although the two methods handle different statistical models, the behavior is much similar, and they all need not special model selection. However, the vague prior may cause an improper posterior [30,31]. Ter Braak et al. [32] proposed to use the prior  with a small value of **δ **but not the extreme value 0 to avoid generating improper posterior, while this extreme value was just used in [17] and my researches. I also attempted to set **δ **= 0.0001 and other values and did several times of experiments, and the results at **δ **= 0.0001 were essentially the same as those at **δ **= 0 (the results were not shown).

Because there are no close forms for the variance components, the M-H algorithm is always used to update them. There are two kinds of M-H algorithm, and one is the RWM-H, in which the new proposal value is conditionally sampled on the old one; the other is called independent Metropolis-Hastings algorithm (IM-H), and the proposed value is independently sampled on the old one. Generally, the RWM-H is more efficient than the IM-H, because in the RWM-H the proposal values may automatically reach their main support region in the iterations. Another advantage of the RWM-H is that the estimate of QTL variance for zero-effect QTL may be gradually converged to zero. However, the special shrinkage character is hardly held by the IM-H algorithm because it is usually low probability that the values of the proposal variance close to zero infinitely. Hence in this article I use a special RWM-H to update QTL variance, which is also another key to my method.

The size of the tuning parameter may influence the efficiency of the RWM-H algorithm. In this method, the degree of freedom ν is equivalent to the tuning parameter, if ν is smaller, the efficiency of the M-H algorithm may decrease due to the low acceptance rate. But if ν is larger, although the acceptance rate increase, it is more difficult for the proposal variance components to explore their posterior distribution. If ν > 200, the chain will be stuck locally and the posterior distribution of variance components will be very difficult to explore, which explains why the shrinkage character is hardly hold by the proposed method when ν >200. In fact, the tuning parameter should be set appropriately, which makes the acceptance rate to be 10~ 40% [33]. Therefore, in my method, the optimal tuning parameter ν should range from 3 to 15 from Figure 4.

I assign a scaled inverted chi-square distribution for polygenic variance and residual variance, which makes the incorporation of the prior information possible, and this has been studied in previous work [21]. Certainly, other priors also can be used and then the formula of acceptance probability should be constructed appropriately.

The polygenic term is excluded in our genome-wide mapping because many QTL with relative large effect are investigated in my simulated study. In practice, the trait may be affected by few QTL with substantial effects and many QTL with minor effects, and then it is necessary to include the polygenic effect in the model.

I proposed a basic method for mapping QTL in variance component models. The method is also important in fine mapping [34-38] in which both linkage information and linkage disequilibrium (LD) information are utilized. If the markers are densely distributed, fine mapping provides an extremely powerful way for QTL mapping. The new method is also convenient to be modified to the simultaneous fine mapping of multiple QTL as long as the IBD matrix is appropriately constructed. Moreover, the method also can be extended to more complicated situation, such as that involving QTL dominant effect and epistatic effect.

I employed the approach of Yi et al. [27] to ascertain the maximum QTL number and found that the method was not very sensitive to the maximum QTL number. Theoretically, the maximum QTL number may be set as any value as long as it is greater than the actual QTL number. The simplest method is to assume that each marker interval contain one QTL, while it increases the computational burden. The appropriate selection of the maximum QTL number will contribute to saving the computational time.

The new developed method is very simple and easy to implement. The computer program is written in FORTRAN language, and it is also compiled into my software "BayesMapQTL.exe" which can be used to analyze simulated data, as well as field data with variable family size. Both program and software are available for request.

## Conclusion

In this research, I proposed a Bayesian shrinkage estimation method for mapping multiple QTL. Different from Xu's (2003) shrinkage method that discriminately estimates QTL substitute effect, my method can shrinkage estimate QTL variance so that it need no special model selection. Simulation experiments show that the proposed method is efficient in simultaneously mapping multiple QTL in outbred populations and may even out-perform the RJMCMC method.

## Appendix

### Joint posterior distribution

Observable parameters include marker information **M**, additive genetic relationship matrix **A**, covariate matrix **X **and phenotypic values ; unobservables include QTL positions **λ **= , model effects  and hyper-parameters . Joint posterior probability of unobservables then can be expressed as(A1)

where,(A2)

and(A4)

### Update QTL position

The proposal position is moved around the old one,  = λ_*j *_+ *d*, where *d *is a random number sampled from uniform distribution with bound -*k*cM and *k*cM, where *k *is a predetermined tuning parameter and equals to 1 for chromosome segment analysis, and 20 for genome-wide scan in my study. When the new position is proposed, the IBD matrix is calculated according to marker information, and then the new position is accepted with probability equal to min(1, *r*),(A5)

The proposal ratio or Hastings ratio *hr*_*p *_= 1 due to the symmetric uniform proposal.
